# Efficacy of the digital health application ORIKO^Ⓡ^ for attention-deficit/hyperactivity disorder (ADHD) in adults: Protocol of a randomized controlled trial^☆^

**DOI:** 10.1016/j.mex.2026.103850

**Published:** 2026-02-28

**Authors:** Lea Schuurmans, Daniel Schöttle, Andreas Reif, Kai G. Kahl, Steffen Moritz, Anne Karow, Martin Lambert, Felix Betzler, Alexandra Philipsen, Anna Baumeister

**Affiliations:** a Department of Psychiatry and Psychotherapy, University Medical Center Hamburg-Eppendorf, Hamburg, Germany; b Department of Psychiatry and Psychotherapy, Asklepios Clinic Harburg, Hamburg, Germany; c Department of Psychiatry, Psychosomatic Medicine and Psychotherapy, University Hospital Frankfurt – Goethe University, Frankfurt am Main, Germany; d Fraunhofer Institute for Translational Medicine and Pharmacology ITMP, Theodor-Stern-Kai 7 60596 Frankfurt am Main, Germany; e Department of Psychiatry, Social Psychiatry and Psychotherapy, Hannover Medical School, Carl-Neuberg-Str. 1 30625 Hannover, Germany; f MiNDNET E-Health Solutions GmbH, Christoph-Probst-Weg 4 20251 Hamburg, Germany; g Charité – Universitätsmedizin Berlin, corporate member of Freie Universität Berlin and Humboldt Universität zu Berlin, Clinic for Psychiatry and Psychotherapy, Charitéplatz 1 10117 Berlin, Germany; h Department of Psychiatry and Psychotherapy, University Hospital Bonn, Venusberg-Campus 1 53127 Bonn, Germany

**Keywords:** Mobile health, Internet-based intervention, Digital health application, App-based therapy

## Abstract

Attention-deficit/hyperactivity disorder (ADHD) often continues into adulthood and affects several million adults in Germany. Many do not receive timely, evidence-based treatment because of long waiting times and limited access to care. Digital health applications offer a low-threshold option to provide guideline-based support. A pilot study of the present app-based program showed encouraging improvements in quality of life and reductions in ADHD symptom severity.

This randomized controlled trial (RCT) aims to confirm and extend the pilot findings. The primary objective is to examine whether the digital intervention improves quality of life in adults with ADHD. Further outcomes include changes in ADHD symptoms, functional impairment, depressive and anxiety symptoms, substance use, general health, health literacy, medication adherence, and user satisfaction.

A total of 380 adults with verified ADHD will be recruited online and via a university hospital. After informed consent and baseline assessment, participants will be randomized 1:1 to immediate access to a 12-week digital unguided program or to care as usual (CAU). Self-report assessments will take place at baseline, 6 weeks, and 12 weeks. Mixed models for repeated measures will be used for the primary analyses, complemented by intention-to-treat and complete-case approaches.

The trial will test the robustness of the previous pilot results and provide evidence on the clinical value of a scalable digital intervention for adult ADHD.

## Graphical abstract

**Figure:**
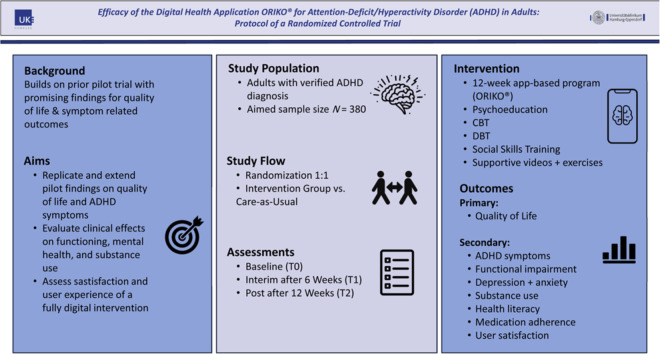

## Specifications table

**Table:** 

**Subject area**	Psychology
**More specific subject area**	*Clinical psychology; digital mental health; adult ADHD treatment*
**Name of your protocol**	*Protocol for a Randomized Controlled Trial Evaluating a Digital Self-Help Intervention for Adults With ADHD*
**Reagents/tools**	*Not applicable. The protocol uses a digital health application (ORIKO^Ⓡ^) and online questionnaires administered via Qualtrics^Ⓡ^. No laboratory reagents or physical equipment are required.*
**Experimental design**	Two-arm parallel randomized controlled trial evaluating a 12-week digital unguided intervention versus care as usual in adults with ADHD, with three assessment time points.
**Trial registration**	*DRKS00037711*
**Ethics**	*The study received approval from the Local Psychological Ethics Committee (LPEK; reference number LPEK-0955) and was conducted in accordance with the ethical standards of the Declaration of Helsinki. Study reporting will adhere to the CONSORT recommendations. While individuals with ADHD contributed to the development of the app, they were not involved in shaping the study design. All participants will give written informed consent prior to enrollment. The trial is prospectively registered in the German Clinical Trials Register (DRKS00037711), where the statistical analysis plan is publicly accessible (*https://www.drks.de/search/de/trial/DRKS00037711*).*
**Value of the Protocol**	• *Aims to confirm and extend findings from a prior pilot study of a digital ADHD intervention* • *Evaluation of effects on quality of life, ADHD symptoms, and broader mental health outcomes* • *Large randomized controlled trial assessing the efficacy of scalable, low-threshold access to care for adults with ADHD*

## Background

Attention-deficit/hyperactivity disorder (ADHD) affects an estimated 1.8–4.7 million individuals in Germany [1]. Around 60 % of those diagnosed continue to experience symptoms into adulthood [2]. Adult ADHD is associated with high rates of psychological and somatic comorbidities, increased health risks, functional impairments, and reduced quality of life [2,3].

Despite its burden, only 10–20 % of affected individuals are correctly diagnosed and treated [3,4]. Among those who receive treatment, the proportion accessing evidence-based care remains low: roughly 30 % receive pharmacotherapy and fewer than 3 % access psychotherapy [2,3]. Barriers include misdiagnosis, limited availability or accessibility, and long waiting times for psychotherapy, often between 6 and 24 months [3,5,6].

Clinical guidelines such as those from the German Society for Psychiatry and Psychotherapy, Psychosomatics and Neurology (DGPPN) [5] and the UK’s National Institute for Health and Care Excellence (NICE) [7] emphasize the need for multimodal, evidence-based interventions that combine psychoeducation, skills training, and, where indicated, pharmacotherapy. Yet, implementation of these recommendations remains inconsistent in routine care.

These gaps highlight the need for low-threshold treatment options. Internet-based interventions (IBI) can address this demand by offering guideline-based interventions, including the recommended psychoeducation and skills training, via smartphone apps. Such approaches may expand access to evidence-based care and reduce treatment delays.

The present protocol builds on a previous pilot trial (DRKS00033320), which provided preliminary evidence that the intervention can improve both quality of life and ADHD symptom burden in adults.

## Objectives

The primary objective of this study is to evaluate the medical benefit of an unguided IBI for adults with ADHD. The main outcome is improvement in self-rated quality of life after completion of the 12-week program. Building on pilot study findings, the trial also aims to confirm the robustness of the intervention’s effect on core ADHD symptoms.

The secondary objectives are to explore reductions in functional impairments, depressive and anxiety symptoms, and substance use, as well as to assess changes in general health status, health literacy, medication adherence, and user satisfaction with the intervention.

## Description of protocol

### Overview

**Fig. 1:**
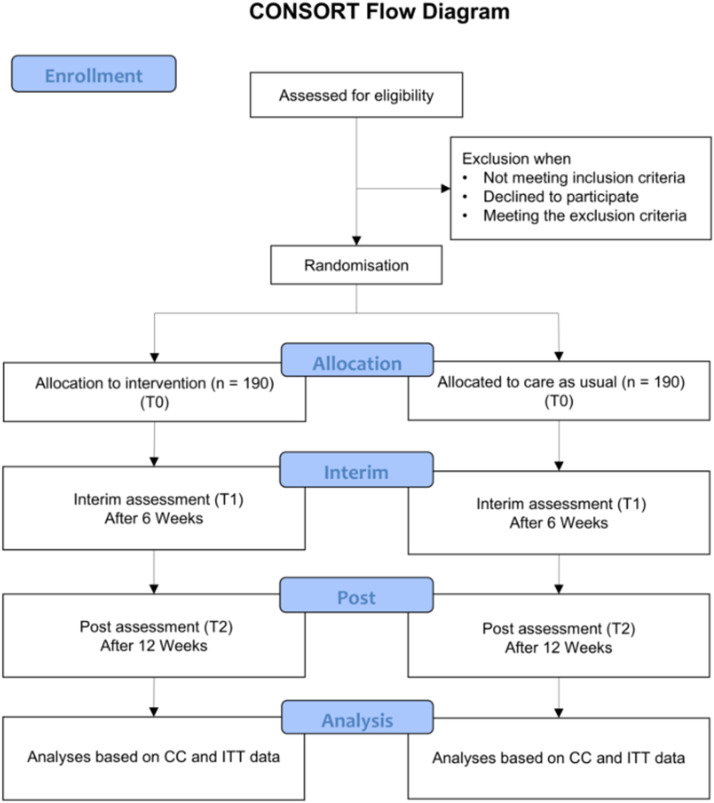
Study Flow.

As illustrated in Fig. 1, the trial follows a two-arm randomized controlled design with three assessments. Participants are randomly assigned to either the intervention group (IG) or a care as usual group (CAU). Assessments take place at baseline prior to the intervention (T0), at 6 weeks (T1), and at 12 weeks (T2).

The IG receives immediate access to the 12-week app-based self-help program (ORIKO^Ⓡ^) following baseline, while the CAU group gains access after completion of the final follow-up at T2. All data are collected online.

### Inclusion and exclusion criteria

**Table:** 

Inclusion criteria	Exclusion criteria

• Minimum age of 18 years • Diagnosis of ADHD according to ICD-10 or DSM-5, documented with date and diagnosing physician/therapist/institution (e.g., medical report) • Access to a smartphone or tablet • Availability for the 12-week study period • Sufficient knowledge of the German language (speaking, reading, and writing) • Current impairment due to ADHD, indicated by an ASRS Screener score ≥ 14 • Stable ADHD medication regimen for at least 4 weeks prior to study start (no change in type or dosage)	• Current diagnosis of a severe psychiatric disorder, including: • Severe unipolar depression (ICD-10: F32.2) • Bipolar disorder (ICD-10: F31) • Schizophrenia, schizotypal, or delusional disorder (ICD-10: F2) • Borderline Personality Disorder (F.60.3) • Ongoing alcohol and/or drug dependence (ICD-10: F1x.2) • Current suicidality (past six months) • Ongoing participation in ADHD-specific psychotherapy • Current initiation or adjustment of ADHD-specific pharmacotherapy (e.g., methylphenidate, amphetamine, atomoxetine)

### Recruitment

Recruitment will be conducted via the University Medical Center Hamburg-Eppendorf (UKE). In addition, further recruitment will take place through other sources including email contact with individuals who previously expressed interest in the pilot study (DRKS00033320), referrals by clinicians at the specialized ADHD outpatient clinic of the UKE, online advertisements (e.g., social media and patient organizations such as *ADHS Deutschland e.V.*), and self-initiated contact by interested individuals via a dedicated study email address. The planned recruitment period is three months. Individuals recruited online must submit written proof of their diagnosis before they can access the baseline survey. Participants may withdraw from the study at any time without providing a reason and without any negative consequences for their medical treatment or relationship with healthcare providers. Participation is voluntary and independent of routine care. Although recruitment occurs partly within healthcare settings, treating clinicians are not involved in obtaining consent, and refusal or withdrawal does not affect access to treatment as usual.

### Randomization and blinding

Participants are randomized automatically in a 1:1 ratio (IG vs. CAU) using an algorithm embedded in the online survey platform Qualtrics^Ⓡ^. Randomization occurs after informed consent and completion of the baseline assessment, minimizing allocation bias. Group allocation is disclosed to participants at the end of the baseline survey, as blinding is not feasible: no placebo condition exists, and participants must know whether they receive immediate or delayed access to the app.

Data collection is based entirely on digital self-report questionnaires administered through Qualtrics, eliminating potential assessor bias. Because allocation is automated and outcome scoring is standardized, neither study staff nor data analysis procedures are subject to manipulation.

### Care as usual

The intervention is designed to be delivered within routine healthcare. Participation in the study does not preclude concurrent treatment; therefore, ongoing CAU is permitted. CAU may include, for example, the use of psychotropic medication. Medication use, including type and dosage, will be documented at baseline (T0) and reassessed at both follow-ups (T1 and T2).

### Intervention: ORIKO^Ⓡ^

The ADHD therapy app ORIKO is a digital self-help program developed for adults aged 18 years and older. It is designed to complement standard treatment provided by general practitioners or specialists, with the overarching goal of improving health status in individuals with ADHD. Consistent with guideline recommendations for adults with ADHD [2,3,5,8], the app provides digital psychotherapy based on cognitive-behavioral therapy (CBT), dialectical behavioral therapy (DBT), and social skills training (SST). Psychoeducational modules are also included to enhance knowledge and support self-management. The structured therapy pathway consists of 12 sequential modules developed by clinical experts in ADHD psychotherapy. Content is delivered through short video sequences featuring physicians, psychotherapists, and individuals with ADHD, and is accompanied by exercises that facilitate transfer into everyday life. An overview of the modules can be found in Table 1.

**Table 1:** Overview of intervention modules.

Module Category	Module Title
Introductory	Goal setting, motivation, program overview, general psychoeducation, resource activation
Symptom-focused	Attention and focusing
Symptom-focused	Organization and structure
Symptom-focused	Time perception and management
Symptom-focused	Impulse control
Symptom-focused	Emotional regulation, affective stability
Symptom-focused	Managing motor restlessness
Cross-symptom	Procrastination and avoidance
Cross-symptom	Social interactions and communication
Cross-symptom	Self-care
Cross-symptom	Sleep and recovery
Final	Relapse prevention (long-term maintenance)

Note. Modules are structured into introductory, symptom-focused, cross-symptom, final, and optional categories.

### Procedure and assessments

Potential participants are recruited at UKE or via online outreach and receive detailed study information along with a link to the digital informed consent form and baseline survey (T0). After consent, they complete the questionnaires listed in Table 1. Participants are then randomized (see Randomization and Blinding). Those allocated to the IG receive an access code to the app, while the CAU receives access after the final assessment (T2). Identical surveys are administered at 6 weeks interim (T1) and 12 weeks (T2), with the IG additionally completing the CSQ-8 at T1 and T2. At each assessment, medication status and CAU are recorded. Participants are compensated with an online shopping voucher upon completion of follow-up assessments.

### Measures

An overview of the assessment time points is provided in Table 2.

**Table 2:** Schedule of assessments.

		Baseline (T0)		Interim (T1)	Post (T2)
		Prior to randomization		Week 6	Week 12
		Screening	Assessment		
Informed consent		X			
Sociodemographics*		X			
Self-stigma (ISMI-9)			X	X	X
Experience with psychotherapy		X			
Inclusion/exclusion		X			
Care as usual*			X	X	X
**Outcomes**					
AAQoL			X	X	X
ASRS			X	X	X
CGI-S			X	X	X
PHQ-9			X	X	X
GAD-7			X	X	X
AUDIT-C/DUDIT			X	X	X
HLS-EU			X	X	X
BFIS			X	X	X
MARS-D			X	X	X
CSQ-8				X	X

Notes: 'Sociodemographics' includes information on gender, age, education, nationality, and native language; 'Care as usual' includes information on previous psychotherapy and current medication with psychotropic drugs (including dosage).

### Primary

#### Quality of life

The Adult ADHD Quality of Life Scale (AAQoL) [9] is the primary outcome. This 29-item self-report instrument covers four domains: life productivity, psychological health, life outlook, and relationships, rated on a 5-point Likert scale (1 = not at all/never, 5 = extremely/very often). Internal consistency is rated excellent (Cronbach’s α = 0.93).

### Secondary

#### ADHD symptoms

The Adult ADHD Self-Report Scale (ASRS) [10] includes 18 items scored from 0 (never) to 4 (very often). It yields a total score (0–72) and two subscales: inattention and hyperactivity/impulsivity (0–36 each). Internal consistency is considered good (Cronbach’s α = 0.88).

#### Functional impairment (global)

ADHD-related impairment in daily life is assessed with an adapted item from the Clinical Global Impression – Severity scale (CGI-S) [11]. Participants rate their impairment on a 7-point Likert scale from not at all impaired (1) to severely impaired (7).

#### Depressive symptoms

The Patient Health Questionnaire-9 (PHQ-9) [12] measures depressive symptoms over the past two weeks with nine items rated from 0 (not at all) to 3 (nearly every day). Total scores range from 0 to 27 and can be categorized into minimal, mild, moderate, and severe depression. Reliability is high (Cronbach’s α = 0.89).

#### Anxiety symptoms

The Generalized Anxiety Disorder Scale-7 (GAD-7) [13] assesses anxiety symptom frequency over the past two weeks with seven items rated from 0 (not at all) to 3 (nearly every day). Internal consistency is excellent (Cronbach’s α = 0.92).

#### Substance use

Alcohol use is measured with the 3-item Alcohol Use Disorders Identification Test -Consumption (AUDIT-C) [14]. The AUDIT-C demonstrated excellent internal consistency (Cronbach’s α = 0.98) [15].

Drug use is screened with the 11-item Drug Use Disorders Identification Test (DUDIT). The DUDIT showed good to excellent internal consistency (Cronbach’s α = 0.80) [16].

Both are scored on 5-point Likert scales and are based on ICD-10 and DSM-5 diagnostic criteria.

#### Health literacy

Perceived health literacy is assessed with the European Health Literacy Survey Questionnaire (HLS-EU) [17]. Sixteen items measure how easy or difficult it is to access, understand, evaluate, and apply health-related information (1 = very easy, 4 = very difficult). The internal consistency is good (Cronbach’s α = 0.88) [18].

#### Psychosocial functioning

Broader functioning is measured with the Barkley Functional Impairment Scale (BFIS) [19]. This validated, norm-referenced self-report tool assesses impairment across 15 life domains. Each item is rated on a 10-point scale (0 = not at all, 8–9 = severe). The BFIS showed excellent internal consistency (Cronbach’s α = 0.97) [20].

#### Medication adherence

For participants currently using ADHD medication, adherence is assessed with the Medication Adherence Report Scale – German version (MARS-D) [21]. The five items are scored on a 5-point scale from never (5) to always (1). The MARS-D has satisfactory internal consistency (Cronbach’s α = 0.60–0.69).

### Additional measures and assessments

#### User satisfaction

Satisfaction with the digital intervention is measured at T2 using the Client Satisfaction Questionnaire-8 (CSQ-8) [22,23]. The eight items are rated on a 4-point scale, with higher total scores reflecting greater satisfaction. The CSQ-8 shows good psychometric properties (α = 0.88–0.92) [24].

#### Self-stigma

Internalized stigma is assessed with the 9-item short form of the Internalized Stigma of Mental Illness Scale (ISMI-9) [25]. Items are rated on a 4-point Likert scale (strongly disagree to strongly agree). The short form demonstrates strong reliability and validity comparable to the full version.

#### Intervention adherence

Engagement with the digital program is tracked descriptively using log-file data (frequency of access and number of completed modules).

### Statistical analyses

#### Sample size

The sample size was calculated in G*Power (version 3.1) [26] for an independent-samples *t*-test targeting the primary endpoint. Assuming a medium effect (Cohen’s *d* = 0.40), two-tailed α = 0.05, and 1−β (power) = 0.90, the required total sample is *N* = 266 (133 per group). Because digital interventions commonly face attrition around 25 % (e.g., [27]), we inflated the sample by 30 %. The resulting recruitment target is *N* = 380 (*n* = 190 per group). This calculation is conservative relative to the mixed-models approach planned for the primary analysis. All analyses will be performed using IBM SPSS^Ⓡ^ Statistics 31 and R 4.4.3 [28].

### Analysis populations

#### Complete cases (CC) analysis

The complete case analysis (CC analysis) includes all data sets from participants for whom both baseline and post-intervention data are available.

#### Intention-to-Treat (ITT) analysis

The intention-to-treat (ITT) analysis includes data sets from all randomized participants. The ITT analysis will be conducted as the primary analysis.

#### Descriptive analyses

Sociodemographic and clinical variables will be summarized using means and standard deviations for continuous variables and absolute frequencies and percentages for categorical variables.

#### Hypotheses

The primary hypothesis is that participants of the IG will show greater improvement in quality of life, as measured by the AAQoL, at 12 weeks (T2) compared to participants in CAU. Secondary hypotheses are that, at 12 weeks (T2), participants in the IG will demonstrate greater reductions in ADHD symptom severity (ASRS), global functional impairment (CGI-S), depressive symptoms (PHQ-9), anxiety symptoms (GAD-7), substance use (AUDIT-C, DUDIT), and greater improvements in health literacy (HLS-EU), daily functioning (BFIS), and medication adherence (MARS-D) compared to CAU.

#### Efficacy analyses

Primary efficacy will be evaluated at 12 weeks (T2), defined as the primary endpoint, using mixed models for repeated measures (MMRM), a likelihood-based approach suitable for longitudinal data and missingness under the missing-at-random assumption [29,30]. Models will include fixed effects for group, time, and group × time interaction, with baseline values entered as covariates. A random intercept for participants and an unstructured covariance matrix will be specified. Degrees of freedom will be estimated using the Kenward–Roger approximation.

#### Sensitivity analyses

In addition to the main MMRM, ITT analyses using the Jump to Reference (J2R) method [31] and CC analyses will be conducted. For these, change scores from baseline (T0) to follow-up (T2) will be analyzed via analysis of covariance (ANCOVA) with baseline as covariate [32,33]. J2R reflects the treatment policy estimand as defined by the EMA [34].

#### Clinical relevance

Outcomes relevant to medical benefit will be interpreted against established Minimal Clinically Important Difference (MCID) thresholds: ≥8-point increase on the AAQoL [35] and ≥30 % reduction on the ASRS [36].

#### Multiplicity control

A hierarchical testing procedure will be applied. Significance will be declared at each step only if the *p*-value < 0.05 and all preceding tests are significant. The testing order is:

1. Quality of life (AAQoL)
2. ADHD symptoms (ASRS)
3. Global functional impairment (CGI-S)
4. Depressive symptoms (PHQ-9)
5. Anxiety symptoms (GAD-7)
6. Substance use (AUDIT-C)
7. Substance use (DUDIT)
8. Health literacy (HLS-EU)
9. Daily functioning (BFIS)
10. Medication adherence (MARS-D)

#### Exploratory analyses

Subgroup analyses will be conducted for primary and secondary outcomes by age (≤65 vs. >65 years), gender (male/female/diverse), current medication status (yes/no), and app use (dichotomized at median module completion). In addition, exploratory analyses will examine changes in perceived self-stigmatization, as measured by the ISMI-9.

### Patient safety

At each assessment (baseline and follow-ups), participants are provided with the contact details of the study team and may reach out at any time in case of symptom deterioration, unexpected negative experiences or study-related questions. Adverse events are not assessed using a standardized questionnaire. Instead, potential adverse effects are monitored through participant-initiated contact with the study team and are documented as part of ongoing study monitoring. Participants remain fully embedded in the regular healthcare system in Germany, and participation does not restrict access to treatment as usual, including mental health care. Given the low-risk, self-guided nature of the digital intervention and the absence of medical procedures, no independent data monitoring committee was established.

### Comparison to prior work

Previous research on digital interventions for ADHD in adults remains limited. While pilot trials and small-scale feasibility studies have suggested improvements in quality of life, ADHD symptoms, and functional outcomes, few randomized controlled trials have been conducted to date. The present study builds on this early evidence by implementing a larger RCT with a comprehensive assessment strategy, thereby extending prior work and addressing the need for robust data on the clinical utility of digital health applications in ADHD.

### Strengths

Key strengths of the study include its randomized controlled design, preregistration, and use of validated outcome measures across multiple domains of functioning. Another strength is that ADHD diagnoses are verified by trained study staff prior to enrollment, ensuring diagnostic accuracy and reducing the risk of including ineligible participants. The entirely digital recruitment, intervention, and assessment process supports feasibility, scalability, and broad accessibility. In addition, both intention-to-treat and sensitivity analyses are planned, strengthening the robustness of the findings.

## Protocol validation

De-identified data generated during the study will be made available upon reasonable request to the corresponding author, in line with data protection regulations and institutional policies.

## Limitations

Some limitations should be considered. Participant blinding is not feasible due to the nature of the digital intervention and the waitlist control with CAU design, which may introduce expectancy effects. Outcomes are assessed exclusively via self-report, which may be subject to reporting bias. In addition, recruitment and adherence may be challenging, as digital interventions for ADHD can be associated with higher attrition rates. Although the waitlist design ensures that all participants eventually receive access to the intervention, it may raise ethical considerations related to delayed treatment for participants assigned to the control condition. This concern is mitigated by the relatively short waiting period, and the fact that participation does not restrict access to care as usual. Finally, the fully digital and self-guided format limits personal contact, which may affect engagement for some participants.

## Conclusion

This protocol describes a randomized controlled trial designed to evaluate the efficacy and clinical relevance of a digital self-help intervention for adults with ADHD. By building on promising pilot findings (DRKS00033320, publication in prep.) and applying a rigorous confirmatory design with predefined endpoints and statistical procedures, the study aims to provide robust evidence on the intervention’s effects on quality of life and symptom burden and further explore the potential for other related health domains. The fully digital and scalable format reflects current developments in mental health care and may contribute to improving access to guideline-based support for adults with ADHD. The results of this trial will inform future implementation and further development of digital interventions in routine care.

## Data management

Study data are collected and processed exclusively for scientific purposes. Data ownership lies with the application developer, while data storage and processing are carried out by the study site in compliance with applicable data protection regulations. Data are stored on secure servers of the UKE with access restricted to authorized study personnel. Personal contact information is stored separately from study data and processed in a pseudonymized manner. Study data will be retained for ten years after study completion in accordance with institutional and legal requirements. Any further scientific use beyond the present study will require appropriate ethical approval and will be limited to pseudonymized data. Participants are informed about data processing, storage, retention periods, and their rights in the study-specific data protection information.

## CRediT author statement

Lea Schuurmans: Conceptualization, Data curation, Formal analysis, Investigation, Methodology, Project administration, Validation, Visualization, Writing - Original Draft, Writing - Review & Editing; Daniel Schöttle: Writing - Review & Editing, Methodology, Project administration; Andreas Reif: Writing - Review & Editing, Methodology, Project administration; Kai G. Kahl: Writing - Review & Editing, Methodology, Project administration; Steffen Moritz: Writing - Review & Editing, Methodology, Supervision, Project administration; Anne Karow: Writing - Review & Editing, Software, Resources, Methodology, Project administration; Martin Lambert: Writing - Review & Editing, Software, Resources, Methodology, Project administration; Felix Betzler: Writing - Review & Editing, Methodology, Project administration; Alexandra Philipsen: Writing - Review & Editing, Methodology, Project administration; Anna Baumeister: Conceptualization, Data curation, Formal analysis, Investigation, Methodology, Project administration, Supervision, Validation, Visualization, Writing - Original Draft, Writing - Review & Editing

## Declaration of generative AI and AI-assisted technologies in the manuscript preparation process

During the preparation of this work, the authors used institutional access through the University of Hamburg to OpenAI API (ChatGPT, model GPT-4 omni mini) to enhance the clarity and structure of text passages. After using this tool, the authors reviewed and edited the content as needed, and they take full responsibility for the content of the published article.

## Declaration of competing interest

The authors declare the following financial interests/personal relationships which may be considered as potential competing interests:

**AB** and **SM** have received honoraria for lectures and/or serving on advisory boards from Boehringer Ingelheim. **DS** has received honoraria as a lecturer, served as an advisory board member and/or received research funding from 10.13039/100016040Takeda and Medice Pharmaceuticals. He is part of the ORIKO scientific advisory board and was involved in the development of the program. **AR** has received honoraria for lectures and/or serving on advisory boards from 10.13039/501100024879Janssen, 10.13039/100001003Boehringer Ingelheim, COMPASS, SAGE/Biogen, 10.13039/100013410LivaNova, Medice, Shire/Takeda, 10.13039/100030732MSD, and Cyclerion. AR has also received research grants from Medice and 10.13039/501100024879Janssen. **KGK** has received funding from the German Federal Ministry of Education and Research (BMBF), JobCenter Hannover, HannoverPLUS Foundation, and German Research Foundation (DFG). He reports serving on advisory boards for Takeda, Servier, Eli Lilly, and Johnson & Johnson. He has given lectures sponsored by Takeda, Eli Lilly, Idorsia, and Johnson & Johnson. **AK** has been a consultant and/or advisor to or has received honoraria during the past three years from Lilly Deutschland GmbH, Janssen Cilag GmbH, Otsuka Pharma GmbH, Laboratorios Farmacéuticos Rovi, and Takeda Pharma Vertrieb GmbH & Co. KG and has shares in the MiNDNET AG and GmbH. **ML** holds shares in, and serves as the Chief Medical Officer (CMO) of, Mindnet E-Health AG. He has received honoraria as a lecturer and/or research funding from Medice Pharmaceuticals. He was involved in the development of the program. He has been a consultant and/or advisor to or has received honoraria during the past three years from Janssen Cilag GmbH, Otsuka Pharma GmbH, Laboratorios Farmacéuticos Rovi, Takeda Pharma Vertrieb GmbH & Co. KG, and TEVA Pharma and has shares in the MiNDNET AG and GmbH. **AK** and **ML** were involved in the conceptualization of the study but had no role in data collection and analysis, interpretation of the results, or decision to publish. **FB** has received honoraria as a lecturer, served as an advisory board member and/or received research funding from 10.13039/100031585Takeda and Medice Pharmaceuticals. He is part of the ORIKO scientific advisory board and was involved in the development of the program. **AP** has received funding from the German Federal Ministry of Education and Research, Horizon2020, Medice, German Research Foundation (DFG), and 10.13039/100014461NIHR; she has received honoraria as a lecturer and she serves on advisory boards for Takeda, Medice, and Boehringer; has delivered lectures sponsored by Medice and 10.13039/100016040Takeda; and is the author of books and articles on psychotherapy.

## Data Availability

No data was used for the research described in the article.
